# Supplementary material for: A mechanism for the extension and unfolding of parallel telomeric G-quadruplexes by human telomerase at single-molecule resolution
Source: eLife. 2020 Jul 29;9:e56428. doi: 10.7554/eLife.56428 (PMC7426096; doi:10.7554/eLife.56428)
Supplement: Supplementary file 1. [file elife-56428-supp1.docx]

Supplementary material for:

A mechanism for the extension and unfolding of parallel telomeric G-quadruplexes by human telomerase at single-molecule resolution.

**Bishnu P. Paudel, Aaron Lavel Moye, Hala Abou Assi, Roberto El-Khoury, Scott B. Cohen, Jessica K. Holien, Monica L. Birrento, Siritron Samosorn, Kamthorn Intharapichai, Christopher G. Tomlinson, Marie-Paule Teulade-Fichou, Carlos González, Jennifer L. Beck, Masad J. Damha, Antoine M. van Oijen, and Tracy M. Bryan**

**Supplementary Table 1:** Oligonucleotides used in this study

| **Name** | **Sequence (5' to 3')** | **Extinction coefficient (L*M^-1^cm^-1^)** |
| --- | --- | --- |
| 22G0 | AGGGTTAGGGTTAGGGTTAGGG | 228,500 |
| 22G3 | A(fG)GGTTA(fG)(fG)GTTA(fG)(fG)GTTA(fG)GG | 228,500 |
| 22G0-tail | TGGCGACGGCAGCGAGGCTAGGGTTAGGGTTAGGGTTAGGG | 407,300 |
| 22G3-tail | TGGCGACGGCAGCGAGGCTA(fG)GGTTA(fG)(fG)GTTA(fG)(fG)GT^AlexaFluor555^TA(fG)GG | 407,300 |
| 647-Strand2 | GCCTCGCT^AlexaFluor647^GCCGTCGCCA-Spacer18-Biotin | 192,600 |
| 7GGT | TTAGGGT | 69,800 |
| Bio-sp-7GGT | Biotin-Spacer18-TTAGGGT | 69,800 |
| 647-7GGT | Spacer18-TT^AlexaFluor647^AGGGT | 69,800 |
| 555-7GGT | Spacer18-TT^AlexaFluor555^AGGGT | 69,800 |
| Bio-L-18GGG | Biotin-CTAGACCTGTCATCATTAGGGTTAGGGTTAGGG | 325,000 |
| RNA.10C | CUAACCCUAA | 98,200 |
| DNA.10C | CTAACCCTAA | 96,600 |

(fG) = 2'F-araG
